# Gene flow from an adaptively divergent source causes rescue through genetic and demographic factors in two wild populations of Trinidadian guppies

**DOI:** 10.1111/eva.12356

**Published:** 2016-02-04

**Authors:** Sarah W. Fitzpatrick, Jill C. Gerberich, Lisa M. Angeloni, Larissa L. Bailey, Emily D. Broder, Julian Torres‐Dowdall, Corey A. Handelsman, Andrés López‐Sepulcre, David N. Reznick, Cameron K. Ghalambor, W. Chris Funk

**Affiliations:** ^1^ Kellogg Biological StationDepartment of Integrative BiologyMichigan State UniversityHickory CornersMIUSA; ^2^ Department of BiologyColorado State UniversityFort CollinsCOUSA; ^3^ Graduate Degree Program in EcologyColorado State UniversityFort CollinsCOUSA; ^4^ Department of Fish, Wildlife, and Conservation BiologyColorado State UniversityFort CollinsCOUSA; ^5^ Lehrstuhl für Zoologie und EvolutionsbiologieDepartment of BiologyUniversity of KonstanzKonstanzGermany; ^6^ CNRS UMR 7618Institute of Ecology and Environmental Sciences of Paris (iEES)Université Pierre et Marie CurieParisFrance; ^7^ Department of Biological and Environmental SciencesCenter of Excellence in Biological InteractionsUniversity of JyväskyläJyväskyläFinland; ^8^ Department of BiologyUniversity of CaliforniaRiversideCAUSA

**Keywords:** capture‐mark‐recapture, demographic rescue, fitness, gene flow, genetic rescue, hybridization, *Poecilia reticulata*, population growth

## Abstract

Genetic rescue, an increase in population growth owing to the infusion of new alleles, can aid the persistence of small populations. Its use as a management tool is limited by a lack of empirical data geared toward predicting effects of gene flow on local adaptation and demography. Experimental translocations provide an ideal opportunity to monitor the demographic consequences of gene flow. In this study we take advantage of two experimental introductions of Trinidadian guppies to test the effects of gene flow on downstream native populations. We individually marked guppies from the native populations to monitor population dynamics for 3 months before and 26 months after gene flow. We genotyped all individuals caught during the first 17 months at microsatellite loci to classify individuals by their genetic ancestry: native, immigrant, F_1_ hybrid, F_2_ hybrid, or backcross. Our study documents a combination of demographic and genetic rescue over multiple generations under fully natural conditions. Within both recipient populations, we found substantial and long‐term increases in population size that could be attributed to high survival and recruitment caused by immigration and gene flow from the introduction sites. Our results suggest that low levels of gene flow, even from a divergent ecotype, can provide a substantial demographic boost to small populations, which may allow them to withstand environmental stochasticity.

## Introduction

The fate of wild populations exposed to environmental variation is determined by an interplay between genetic variation and demography (Lande 1988). Small populations are vulnerable to the loss of genetic variation due to drift and inbreeding, which in turn may cause population decline and an inability to adapt to changing environments (Keller and Waller 2002; Spielman et al. 2004). A lack of genetic diversity has been implicated in many population and species extinctions (Newman and Pilson 1997; Saccheri et al. 1998; Fagan and Holmes 2006). Given that *de novo* mutations may arise too slowly to benefit genetically imperiled populations (Lande 1980), one way to reconnect recently fragmented small populations, or infuse genetic variation into inbred populations, is through managed movement of individuals or gametes (Weeks et al. 2011; Aitken and Whitlock 2013; Carlson et al. 2014). Ideally, gene flow caused by assisted migration would result in genetic rescue, defined as an increase in population growth owing to the infusion of new alleles by more than the amount attributed to the demographic input alone (Tallmon et al. 2004). Genetic rescue presents a possible temporary solution, albeit contentious, for curtailing the loss of imperiled populations (Edmands 2007; Whiteley et al. 2015), and has successfully caused the rebound of high profile species like the Florida panther (Johnson et al. 2010b) and the Rocky Mountain bighorn sheep (Hogg et al. 2006). However, use of this management strategy remains controversial and perhaps under‐utilized due to concerns that outbreeding depression will cause reduced fitness of offspring between genetically divergent parents (Hufford and Mazer 2003; Frankham et al. 2011).

Predicting the success of genetic rescue as a management tool remains a challenge, largely due to the poor ability to predict the fitness effects of gene flow (Garant et al. 2007). Theory predicts that gene flow can boost fitness when recipient populations are small and inbred (Slatkin 1985), but depending on the strength and direction of selection in different environments, excessive gene flow may homogenize populations, constrain local adaptation, and ultimately reduce fitness (Garcia‐Ramos and Kirkpatrick 1997). While some studies have shown that phenotypic divergence is often reduced between highly connected populations (Lu and Bernatchez 1999; Hendry and Taylor 2004; Nosil and Crespi 2004), other studies have documented adaptive divergence in the face of high gene flow (Nosil et al. 2008, 2009; Fitzpatrick et al. 2015; Moody et al. 2015), demonstrating that selection can overcome homogenizing effects of gene flow. We still lack an understanding of the net effects of gene flow on fitness, particularly when immigrants are from an adaptively divergent source but the recipient population is small, and potentially inbred.

Despite its practical importance, rigorous tests of genetic rescue in wild populations are rare (Whiteley et al. 2015). Most studies are limited to comparing fitness components between locally adapted individuals and first‐ or second‐generation hybrids, while long‐term genetic rescue studies are uncommon (but see Madsen et al. 2004). Multi‐generational studies in the wild are crucial because an increase in individual fitness measured in one or several traits in the lab may not reflect the outcome of gene flow on demography for several reasons. First, successful genetic rescue ultimately depends on population growth rate and not individual fitness components (Whiteley et al. 2015). Second, theory predicts that the effects of gene flow will vary over time (Dobzhansky 1948). For example, a study on marine copepods showed that heterosis in F_1_ hybrids was followed by a decrease in fitness in later generations due to the breakdown of co‐adapted gene complexes (Edmands 1999). Finally, the effects of gene flow on fitness can be very different under laboratory rather than natural conditions (Armbruster and Reed 2005). In the wild, environmental stress can exacerbate the effects of inbreeding depression and magnify heterosis following gene flow (Keller and Waller 2002). Furthermore, maladapted immigrants may contribute little to the breeding population (Sakai et al. 2001), as often documented when hatchery reared individuals are used to supplement small native populations (Araki et al. 2008; Fitzpatrick et al. 2014a).

In this study we took advantage of recent introduction experiments of Trinidadian guppies (*Poecilia reticulata*) in the wild to overcome the above limitations. Specifically, we tested the initial and sustained effects of gene flow between populations of guppies locally adapted to streams with different predator regimes. Guppies adapted to predators were introduced upstream of naturally occurring populations in headwater streams lacking most predators. These translocation experiments were designed by D. Reznick and colleagues to study eco‐evolutionary feedbacks in rapidly adapting populations (Travis et al. 2014). Two of these introductions were conducted upstream from native populations of guppies, and thus we expected unidirectional, downstream immigration and gene flow to occur. Furthermore, native populations of guppies isolated in headwater tributaries are typically small and genetically depauperate (Barson et al. 2009; Willing et al. 2010; Baillie 2012), and thus provided a model for endangered populations that are fragmented and potentially inbred.

We tested the demographic and genetic consequences of gene flow from a divergent immigrant source on native populations by tracking changes in genetic diversity, population size, and population vital rates (survival and recruitment) over multiple generations. This allowed us to assess whether gene flow from a divergent source results in an overall reduction or increase in fitness at the population level. We could also determine whether changes in population size were caused only by demographic factors, genetic factors, or both. If population growth were caused only by high survival and recruitment of immigrants and pure immigrant offspring, demographic but not genetic rescue would be invoked (Brown and Kodric‐Brown 1977). Whereas if hybrid individuals also contributed to an increase in vital rates and population size relative to pure native individuals, we could determine that genetic rescue was involved. Comparing the relative success of different hybrid groups could highlight specific mechanisms involved with rescue. For example, high F_1_ fitness would point to heterosis, but reduced fitness in later generations of hybrids would suggest that recombinant genotypes suffer from outbreeding depression.

## Methods

### Experimental set‐up in the wild

Trinidadian guppies (*P. reticulata*) are a model system in evolutionary ecology that has provided some of the best evidence for rapid adaptation in response to divergent selection (Reznick et al. 1990; Reznick 1997; Magurran 2005). Waterfall barriers found throughout streams of the Northern Range Mountains of Trinidad limit upstream dispersal and result in simple fish communities in headwater tributaries, with increasing diversity in lower elevation and high‐order rivers (Gilliam et al. 1993). Guppies in low elevation streams below waterfalls coexist with a suite of fish that prey on guppies, while most of these predators are excluded from streams at higher elevations. Throughout independent drainages across Trinidad, guppies in high predation (HP) versus low predation (LP) sites show adaptive differences in life history (Reznick and Endler 1982), behavior (Seghers 1974), color (Endler 1980), and morphology (Alexander et al. 2006) that diverge mostly in parallel patterns across predation regime. Additionally, guppy populations in upland LP environments tend to be isolated and genetically depauperate (Crispo et al. 2006; Barson et al. 2009; Baillie 2012). Thus, in our system gene flow from an originally divergent source could either reduce fitness of recipient populations through outbreeding depression, or increase fitness through demographic and genetic factors.

**Figure 1:**
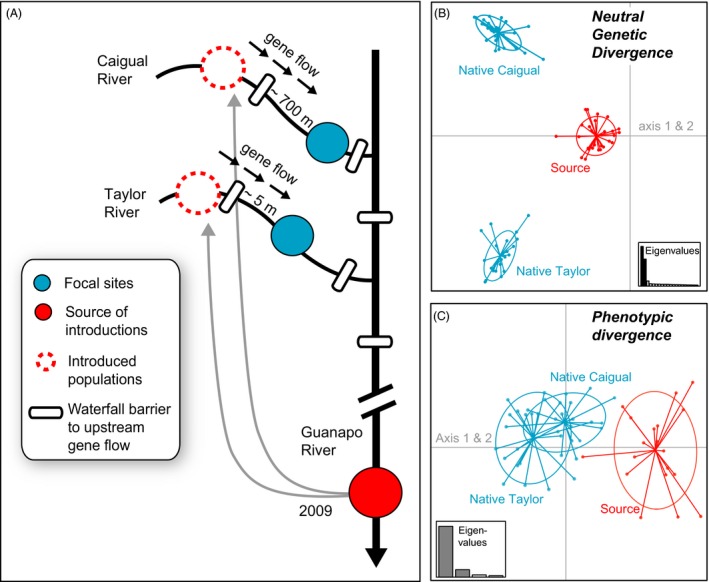
(A) Schematic of the introduction scenario that allowed us to test the effects of gene flow from guppies that originated from an adaptively divergent source population (red) into two native populations (blue). (B) Principal components analyses using microsatellite data highlights initial genetic divergence between the native populations (blue) and the source of the introductions (red). (C) Principal component analyses using phenotypic traits highlights initial phenotypic divergence between native populations and the source of the introductions. Traits included in this analysis were male life history and body shape traits from data published in Fitzpatrick et al. (2015).

We began monitoring two native guppy populations of LP sites in January 2009. Three months later the abovementioned introduction experiment (Travis et al. 2014) was initiated when 150 individuals (75 females, 75 males) descended from a HP locality were introduced into each stream reach upstream of our two study sites that were previously guppy‐free (Fig. 1A). Due to waterfall barriers limiting upstream movement, gene flow was unidirectional from the upstream‐introduced populations into our downstream focal sites. At the onset of the upstream experiment, immigrants were genetically distinguishable (Fig. 1B) and phenotypically divergent (Fig. 1C; Torres‐Dowdall et al. 2012; Fitzpatrick et al. 2015) from our study populations.

### Monitoring of wild populations

Our study sites were located within the Taylor and Caigual Rivers: two neighboring tropical headwater streams from the Guanapo watershed in the Northern Range Mountains of Trinidad. Stream reaches sampled in the Taylor (240 m long) and the Caigual (80 m long) were chosen because they included the upstream extent of native guppies prior to introductions and were bound on either end by waterfalls, thereby preventing upstream movement. Due to the location of waterfall barriers, overall distance between our study sites and the introduction sites differed between streams (Taylor, 5 m; Caigual, 700 m).

Every month from January 2009 to June 2011 (with the exception of April 2009), we recorded every individual captured that had a standard length over 14 mm. We therefore sampled a total of 29 occasions over 30 months, three of which were before upstream introductions in March 2009. Unmarked individuals were given a unique mark for future identification. Guppies were caught using a combination of butterfly nets, hand nets, and minnow traps. We recorded the location of all pools and riffles within the streams in order for fish to be returned to their precise site of capture. Fish were transported to the lab in Nalgene^®^ (Rochester, NY, USA) bottles filled with stream water and held in aerated tanks separated by location and sex. Before processing, fish were anesthetized with a dilute solution of MS‐222 to allow individuals to be marked and photographed. Guppies were marked under a dissecting microscope with visible implant elastomer tags (Northwest Marine Technologies, Inc., Shaw Island, WA, USA) injected subcutaneously. Each fish was given a unique combination of marks using two or three out of eight discrete marking sites, and 12 possible colors. Concurrently, an identical capture‐mark‐recapture protocol was conducted in upstream introduction sites (López‐Sepulcre et al. 2013; Travis et al. 2014). The two studies used nonoverlapping marking codes so guppies entering our focal sites from the introduction sites could be individually identified as immigrants. However, unmarked immigrants such as juveniles could also enter our focal sites. Three scales were removed from all new (unmarked) recruits each month and dried for DNA extraction. All fish were returned to their capture site 1–2 days after initial capture. Previous capture‐mark‐recapture studies on guppies have demonstrated high recapture probabilities, high mark retention, and low marking mortality using these methods (Reznick et al. 1996).

In total we uniquely marked and monitored 9590 individual guppies throughout 29 capture events (months) between 2009 and 2011. Of these, 4710 were captured in Taylor and 4880 were captured in Caigual. We recaptured 88 individuals in Taylor and seven in Caigual that had originally been marked as part of the upstream introduction experiment, and thus were confirmed immigrants.

### Microsatellite genotyping and genetic analyses

We conducted genetic analyses on all individuals from both streams captured during the first 17 (of 29) months of our study. Although we were limited to 17 months of genetic monitoring due to time and resources, this timeframe captured two consecutive wet and dry seasons and 3–4 guppy generations. We extracted genomic DNA from scale samples using Gentra Puregene Tissue Kits (Qiagen, Venlo, The Netherlands). Individuals were genotyped at 12 microsatellite markers developed for this study (Table S1). Microsatellite development and checks for neutrality are described in Appendix S1. We amplified loci using Qiagen Type‐It Microsatellite Multiplex PCR kits with reactions carried out following the manufacturer's recommended conditions. PCR products combined with HiDi formamide and LIZ size standard (500 GeneScan) were read on an ABI 3730xl automated sequencer (Life Sciences Core Laboratories at Cornell University). Microsatellites were visually scored using the microsatellite plug‐in with GENEIOUS 7.1.7 (Kearse et al. 2012). We scored two positive controls and one negative control on each plate and found low genotyping error rate (<0.5%). In total we genotyped 3298 guppies (1807 from Taylor and 1491 from Caigual) at 12 microsatellite loci.

We evaluated changes in genetic diversity over time by grouping genotypes from all individuals captured in a given month, separated by stream. We calculated heterozygosity using ARLEQUIN 3.0 (Excoffier et al. 2005) and allelic richness in the ‘hierfstat’ package in R (Goudet 2005). We used the Bayesian model‐based approach implemented in NEWHYBRIDS v.1.1 (Anderson and Thompson 2002) to assign each individual to one of six genotype frequency classes: pure native, pure immigrant, F_1_ hybrid, F_2_ hybrid, F_1_ × native backcross, F_1_ × immigrant backcross. We assessed the power of NEWHYBRIDS to correctly assign individuals to genotypic classes by generating datasets of 600 simulated individuals per stream population using HYBRIDLAB 1.0 (Nielsen et al. 2006; see Appendix S1). We analyzed the simulated datasets using NEWHYBRIDS and identified posterior probability thresholds that maximized efficiency and accuracy scores (Figure S1) following the approach of Vähä and Primmer (2006). Optimized thresholds were then applied to the real dataset to determine each individual's genotypic class. Individuals known to have pure native genotypes (i.e., those sampled before the onset of gene flow) and a subset of those with pure immigrant genotypes (i.e., those captured with elastomer codes from introduction sites) were used as reference samples for allele frequency priors. Analyses were run using default settings for 100 000 MCMC iterations with the first 10 000 discarded as burn‐in. We used Jeffreys‐type priors for allele frequencies and mixing proportions. Five MCMC runs beginning from random starting points confirmed consistent convergence. Of 3298 genotyped individuals, 3173 were classified into genetic ancestry groups with high certainty by NEWHYBRIDS.

### Demographic modeling

Individual capture‐mark‐recapture data allowed us to estimate population growth rate (*λ*) and the relative contributions of vital rates that contribute to population growth, apparent survival (*ϕ*) and recruitment (*f*), while accounting for detection probability (Pradel 1996; Nichols et al. 2000). We first tested for temporal changes in population growth rate as an indicator of overall population rescue. For example, a steady decrease in population growth rate over time after the onset of gene flow would be consistent with a negative effect of outbreeding depression, whereas an increase in this parameter over time might suggest demographic rescue, genetic rescue, or both. Population growth rate (*λ*) was estimated using the Pradel model, which is a reverse‐time approach that simultaneously incorporates the contribution of survival (*ϕ*) and recruitment (*f*) on the rate of increase in population size between two periods (Pradel 1996). Recruits are new individuals that enter the population through reproduction and/or immigration. For this first analysis we included all individuals from 29 months of capture‐mark‐recapture data. To determine the factors affecting population growth rate (*λ*), we compared the most complex model, which included an interaction between sex, stream, and month, to all possible model simplifications including all two‐way interactions, single factors, and the constant model. We used a maximum likelihood approach to fit the models and compared among them using Akaike's Information Criterion adjusted for sample size AICc and AICc weights (Burnham and Anderson 2002). Mark‐recapture analyses were carried out using Program MARK v.8.0 (White and Burnham 1999). Variation in detection probability (*P*) was modeled with stream by month interactions (described in Appendix S1).

A second set of models was used to test the role of gene flow on population vital rates (survival and recruitment) and to distinguish between demographic and genetic rescue. If demographic rescue were solely responsible for population growth, we would expect immigrants to have the highest vital rates and the rates of native and hybrid groups to be more or less equivalent. However, if genetic rescue contributed to population growth, we would expect hybrids to show higher relative fitness than native fish. Using capture histories from individuals genotyped and monitored during the first 17 months of the study, we grouped individuals by stream, sex, and genetic classification as determined by the NEWHYBRIDS analysis. We excluded individuals with unknown genetic ancestry (*N* = 125 of 3298 fish) from these analyses. We used the survival and recruitment parameterization of the Pradel model to test the effect of genetic ancestry on vital rates. We implemented a sequential modeling approach where we first fit models using the most general structure for recruitment (*f*) and modeled variation in survival (*ϕ*) with a three‐way interaction of stream, sex, and genetic ancestry, and all simplifications. We then modeled variation in recruitment (*f*) in the same way while using the most general structure (and highest supported) for survival (*ϕ*). We were not able to include interactions between monthly variation and other factors in these models due to small sample sizes for some genetic classes per month. However, our primary goal here was to directly test overall impacts of genetic ancestry on population vital rates and we did not have *a priori* reasons to think that different genetic groups would experience more or less favorable environmental conditions in a given month. Models from each of the Pradel model sets were compared using AIC_c_.

For all analyses we obtained maximum likelihood estimates of parameters from the best‐supported models. We tested for overdispersion of the full dataset using the median‐ĉ method (White and Burnham 1999), and found that there was little (ĉ = 1.36, 95% CI = 1.29–1.42). Detection probability was high in both streams, with averaged monthly estimates in Taylor as 0.83 and 0.86 in Caigual (Table S2; Figure S2). Our high detection probabilities allowed precise estimation of parameters of biological interest (population growth rate, survival and recruitment) and suggest that total number of fish captured each month provides a good proxy for overall population size.

## Results

### Gene flow increased genetic diversity

**Figure 2:**
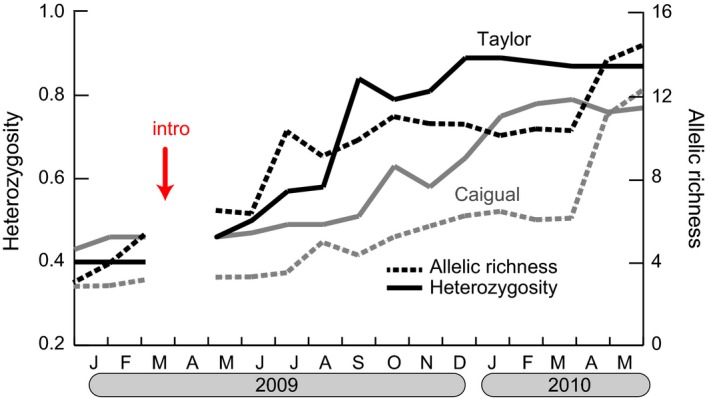
Temporal changes in within‐population genetic diversity following the introductions upstream that occurred in March 2009, as indicated by the red arrow. Solid lines correspond to heterozygosity (scale on left vertical axis) and dashed lines correspond to allelic richness (scale on right vertical axis). Genetic diversity indices were calculated using genotypes from all individuals caught in a given month.

In the months prior to upstream introductions, genetic diversity (heterozygosity and allelic richness) was extremely low within native focal sites of both streams (i.e., average heterozygosity was 0.43 in Caigual and 0.40 in Taylor; Fig. 2). However, monthly averages of genetic diversity increased nearly twofold in both streams following the upstream introduction, consistent with the timing of immigration from the introduction sites. Taylor started with slightly lower levels of heterozygosity and subsequently experienced the most dramatic increase in genetic diversity over time, consistent with the larger number of confirmed marked immigrants detected in this stream.

### Gene flow increased population size

**Figure 3:**
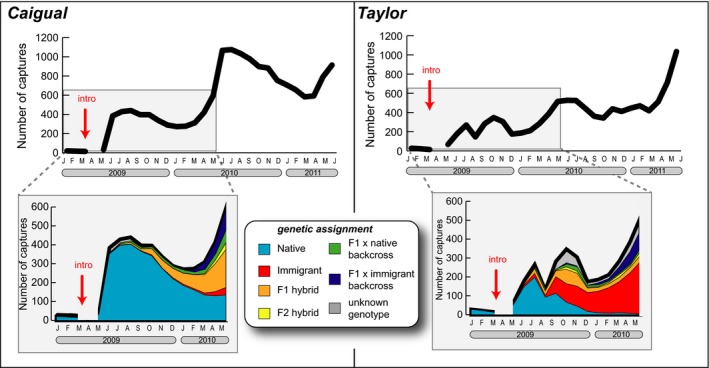
Thick black lines indicate total number of guppies >14 mm captured in each stream over time. Gray boxes correspond to the timeframe in which every individual was genotyped at microsatellite loci for classification into genetic ancestry groups. Colors show the number of individuals in each genetic group caught each month.

Overall high capture probabilities allow us to interpret count data as a relatively good proxy for changes in population size (Figure S2). However, it is important to interpret these data with some caution because differences in detection through time and between streams are not reflected in the overall counts displayed in Fig. 3. Despite substantial seasonal fluctuations, both streams experienced a dramatic increase in population size throughout the course of our study (Fig. 3). Before gene flow we captured fewer than 100 individuals in each stream. By the end of the study the Taylor population reached its highest size of 1035 individuals. The Caigual population reached its highest size of 1075 in July 2010, and we captured 914 guppies on our last sampling occasion. Genetic classifications revealed temporal differences in population dynamics of the different genetic groups in each stream (Fig. 3). Following increases in population size in May and June 2009, the number of pure native genotypes declined in both streams and they were nearly extirpated from Taylor by the end of our genetic monitoring. Concurrently, immigrant genotypes increased to become a large portion of the population in Taylor, while F_1_, F_2_, F_1_ × N, and F_1_ × I hybrids contributed the bulk of the population by May 2010 in Caigual.

### Gene flow influenced vital rates

**Figure 4:**
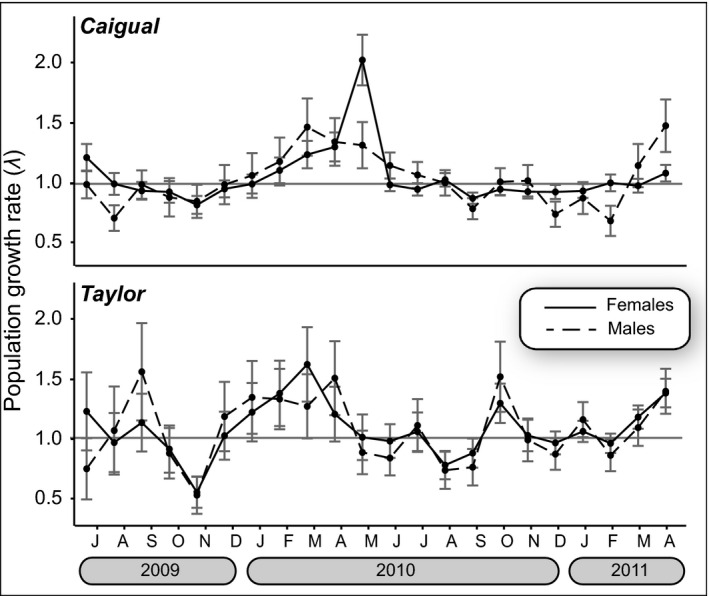
Monthly population growth rate estimates and 95% confidence intervals for males and females from Caigual and Taylor streams throughout the entire duration of study. Estimates are based on capture‐mark‐recapture data and the best‐supported Pradel model (Tables S3).

In our first analysis that included all captured individuals, the full model that included variation in population growth rate by sex, stream, and time interactions was clearly superior, with 100% of the weight of evidence (Table S3). Strong support for this model provides evidence for sex and stream‐specific temporal changes in population growth rate (Fig. 4). Seasonal dynamics seemed to dominate temporal variation in this parameter since *λ* tended to be lowest during rainy season months (June–December) when resources are low (Reznick 1989). For example, populations were generally stable or decreasing during the wet season (*λ *< 1) and increasing during the dry season (*λ *> 1; Fig. 4).

**Figure 5:**
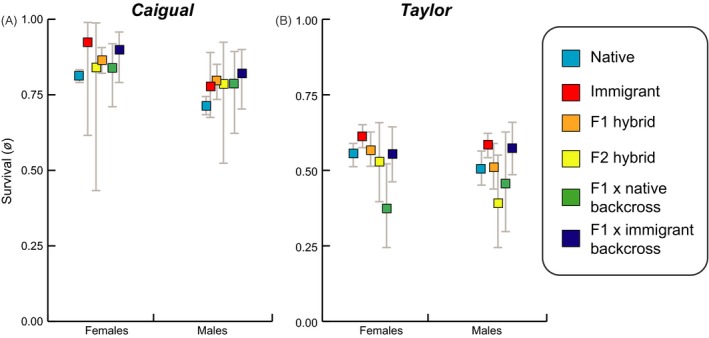
Estimates and 95% confidence intervals of male and female survival probability of different genetic ancestry classifications in Caigual (A) and Taylor (B). All estimates are based on capture‐mark‐recapture data from the first 17 months of the study when all individuals were genotyped and classified into genetic groups. Estimates from the best‐supported Pradel model were plotted (see Tables S4 and S5).

**Figure 6:**
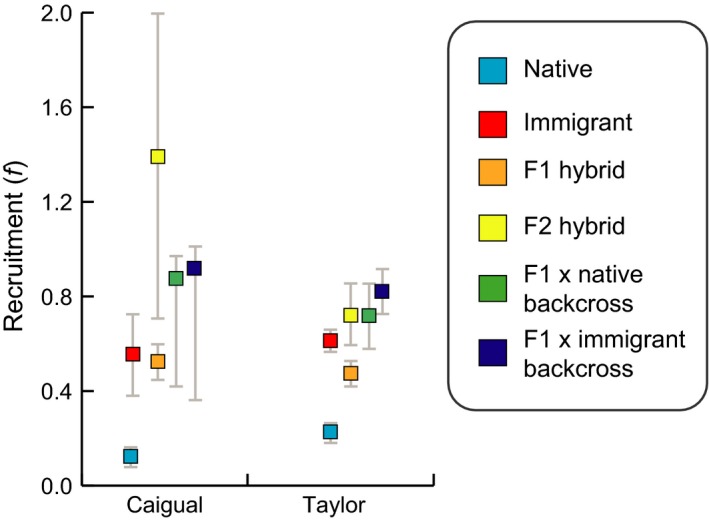
Recruitment estimates and 95% confidence intervals of different genetic ancestry classifications in Caigual and Taylor. All estimates are based on capture‐mark‐recapture data from the first 17 months of the study when all individuals were genotyped and classified into genetic groups. Estimates from the best‐supported Pradel model were plotted (see Tables S4 and S5).

We found support for genetic ancestry explaining variation in both survival (*ϕ*) and recruitment (*f*) in our second set of analyses that only included capture histories from genotyped individuals, but the highest supported models differed between the two vital rates. The full structure (interaction between sex, stream, and genetic ancestry) was the best supported structure for survival (*ϕ*) with 67% of the weight of evidence (Table S4). However, survival estimates did not differ dramatically across genetic groups (Fig. 5A,B) and the next supported survival structure with 37% of the weight of evidence consisted only of the sex by stream interaction. On the other hand, the highest supported structure for recruitment (*f*), with 99% of the weight of evidence, consisted of the genetic group by stream interaction suggesting that sex differences did not explain much of the variation in recruitment, but differences between streams and genetic ancestry did (Table S4). Parameter estimates of recruitment revealed substantial differences in recruitment across genetic classification groups, with native individuals showing lowest recruitment in both streams. Additionally, F_2_ hybrids and both types of backcross hybrids had highest recruitment in both streams, although confidence intervals overlap with immigrants and F_1_ hybrids in Caigual and just immigrants in Taylor (Fig. 6). Parameter estimates from the best supported models are reported in Table S5.

## Discussion

We documented substantial positive effects on population growth that can be attributed to genetic and demographic effects of gene flow (i.e., rescue) in two natural populations. Immigration and subsequent hybridization with genetically and phenotypically divergent individuals led to an overall increase in within‐population genetic variation, abundance, and population vital rates, though dynamic differences were observed between streams and over time. Our results provide a detailed picture of how genetic and demographic rescue can operate in the wild and add to increasing evidence that intraspecific gene flow can be beneficial, even when immigrants are adaptively divergent.

### Evidence for genetic and demographic rescue

Prior to the onset of gene flow, the two native populations in our study were small and genetically depauperate. By the end of our genetic monitoring, spanning 17 months and 3–4 guppy generations, within‐population genetic diversity had more than doubled (Fig. 2). By the end of the full capture‐mark‐recapture study that spanned 29 months and 5–8 guppy generations, population sizes in both streams experienced a 10‐fold increase (Fig. 3). While we acknowledge the limitations associated with the uncontrolled nature of this study and having a single source population and only two recipient populations, several lines of evidence suggest the observed increases in population size resulted from a combination of demographic and genetic factors following immigration and gene flow from divergent source populations.

Genotyping each individual allowed us to distinguish between demographic and genetic rescue. If increases in population size were caused only by demographic rescue, immigrants and their offspring should be the only substantial contributors to the increase in fitness (Brown and Kodric‐Brown 1977). Indeed, the demographic contribution of immigrants is considerable, especially in Taylor where this genotype makes up more than half of the population by May 2010. Predominance of immigrant genotypes in Taylor is likely a result of high migration rates due to the close proximity (~5 m) of focal and introduction sites in this stream, whereas almost 700 m separate these sites in Caigual. But we also found that hybrids contributed substantially to increases in population size in both streams (Fig. 3). F_1_ hybrids were especially successful toward the end of 2009 in Taylor and throughout the study in Caigual suggesting the possible role of heterosis. Additionally, in both streams F_1_ × immigrant backcrosses contributed to population size more than F_1_ × native backcrosses suggesting possible favorable selection for these genotypes.

Estimates of vital rates based on genetic groups revealed minor differences in survival based on genetic ancestry (Fig. 5) but dramatic differences in recruitment between native fish and all other genetic groups (Fig. 6). In fact, later generation hybrids (F_2_, backcrosses) showed highest recruitment in both streams, which suggests that the genetic benefits of crossing persisted beyond first generation hybrids. Genome‐wide SNP data collected from both Caigual and Taylor populations at the end of the study showed that native alleles persisted in both streams, providing additional evidence that hybrid individuals continued to do well and that native alleles were not fully lost from either population (S. W. Fitzpatrick and W. C. Funk, unpublished data). To summarize, the occurrence of genetic rescue is evidenced by the sustained increase in population size and vital rates that can be attributed, at least in part, to the success of the hybrids.

The variation in vital rates that we observed between sexes, streams, and over time is consistent with patterns previously observed in guppies. First, female guppies tend to have higher survival than males (López‐Sepulcre et al. 2013; Fitzpatrick et al. 2014b). Second, variation in abiotic and biotic factors can cause differences in guppy demography even between neighboring streams (Fitzpatrick et al. 2014b). Finally, guppy population sizes in headwater streams fluctuate temporally based on seasonal factors that impact resources and stream flow (Reznick 1989; Grether et al. 2001). Our study began in January, which is typically the start of the dry season in Trinidad, and when guppy population sizes are at their smallest as they have not yet recovered from wet season conditions (Reznick 1989). Our results showed the typical seasonal patterns of decreased population size throughout the wet season (June–December), followed by a recovery during the dry season (January–May), in spite of the onset of immigration and gene flow. Ideally we would have had a longer duration of pre‐gene flow monitoring to track the sizes of native recipient populations during a typical dry season and a control population with similar starting genetic variation and no gene flow. Yet, we found consistent increases in population size throughout multiple seasons monitored in our study (Fig. 3). In other words, even if starting population sizes likely represented the smallest of the year, our study spanned two subsequent wet season cycles in which populations remained well above initial sizes. Additionally, maximum dry season population sizes in 2010 and 2011 were approximately double what they were in 2009 when populations were made up of mostly native individuals.

### Factors that led to rescue over outbreeding depression

Understanding the conditions that underlie opposing fitness outcomes in response to gene flow is a major unresolved problem in evolutionary (Lenormand 2002; Garant et al. 2007) and conservation biology (Edmands 2007). The probability of outbreeding depression is generally determined by the time since isolation of immigrant and recipient populations, the magnitude of environmental differences and resulting level of adaptive divergence between populations, and the level of inbreeding in the recipient population (Frankham et al. 2011). For example, crossing populations with fixed chromosomal differences or those that have been geographically isolated for millions of years is likely to result in outbreeding depression caused by the evolution of postzygotic reproductive barriers such as Dobzhansky‐Muller incompatibilities (Edmands 1999; Coyne and Orr 2004). But at lesser extremes, the extent to which gene flow between adaptively divergent populations reduces overall fitness remains a gray area (Garant et al. 2007). Results from our study lend insight into this question, in part because of the wealth of natural history and genetic information already known about the Trinidadian guppy system. Outbreeding depression was a plausible outcome in the scenario we studied given that the immigrant source population originated from a locality with very different environmental conditions (i.e., higher predation, higher resources and lower guppy density than the recipient locality). Additionally, hybrids could suffer from the breaking apart of co‐adapated gene complexes caused by recombination (Burton et al. 2013). In fact, it is possible that low survival of later generation hybrids observed in the Taylor is a result of this hybrid breakdown (Fig. 5). This finding highlights the importance of understanding the net effects of gene flow on fitness at the population level because not all traits will be impacted in the same way and the ultimate parameter of interest, especially for managers, is how gene flow affects population size over time.

We know from previous work that adaptively divergent guppy populations from the same drainage are not reproductively isolated (Crispo et al. 2006). Features of the guppy mating system such as female preference for novel male color patterns (Eakley and Houde 2004; Olendorf et al. 2006; Hughes et al. 2013) and forced copulation by males (Evans et al. 2003) limit the development of prezygotic reproductive barriers (Labonne and Hendry 2010). And, although selection against migrants is strong when guppies adapted to LP environments are washed downstream or disperse into high‐predation environments (Weese et al. 2011), a low level of downstream gene flow does occur (Barson et al. 2009), which likely prevents accumulation of post‐zygotic reproductive isolation. The introduced populations that provided the source of gene flow in our study, though phenotypically and genetically distinct to a degree, originated from a HP locality in the same drainage as the recipient populations (Fig. 1A) and have experienced low levels of unidirectional downstream gene flow on a contemporary timeframe (Barson et al. 2009; Fraser et al. 2015). Thus, we would not expect these populations to have evolved post‐zygotic reproductive barriers. There is some evidence for post‐mating reproductive isolation and hybrid breakdown when guppies from geographically and genetically distinct drainages are crossed (Ludlow and Magurran 2006; Russell and Magurran 2006). Therefore, we may have observed a different outcome had gene flow originated from a more genetically divergent source.

Conditions of native recipient populations also likely contributed to the observed response to gene flow. Headwater riverine fish populations often exhibit high levels of local inbreeding due to small population sizes and geographic isolation (Fagan 2002). In general, upland guppy populations in LP environments have reduced genetic variation (Crispo et al. 2006; Barson et al. 2009), and inbreeding is known to reduce fitness in guppies (Van Oosterhout et al. 2003; Johnson et al. 2010a). Although we were unable to measure inbreeding depression in our focal populations *per se*, the native populations exhibited extremely low levels of genetic diversity, even when compared to other LP guppy populations throughout Trinidad (Baillie 2012). In addition, the native focal populations showed signs of potential inbreeding depression such as poor health in Taylor (Fitzpatrick et al. 2014b) and overall reduced male coloration compared to guppies from other LP sites (S. W. Fitzpatrick, personal observation). Therefore, fitness benefits from mating with unrelated, immigrant individuals may have been particularly strong if the native populations indeed had high genetic load (Keller and Waller 2002). Even if immigrants were maladaptive for some traits, natural selection acting on the influx of genetic variation following gene flow could increase absolute fitness (Carlson et al. 2014). Additionally, recent work has shown that the fitness of the HP phenotype is superior, even in a LP environment, when populations are at low densities (Bassar et al. 2013). If the native populations we studied were indeed inbred, they may have existed at lower densities than what is typical for these environments, causing them to be more easily invaded by the HP phenotype. Thus, competitive dynamics likely played an important role, and the decline of the native genotype may not have been representative of their trajectory had they not been exposed to competition with hybrids and immigrants.

Characteristics of the immigrants, such as certain life history traits, may have also played a role in determining the demographic success of this group. Guppies adapted to HP environments typically exhibit a fast life history, maturing at a younger age and producing larger broods during shorter intervals than guppies adapted to LP environments (Reznick et al. 1990; Bronikowski et al. 2002; Torres‐Dowdall et al. 2012). Thus, high recruitment of immigrants could have resulted from exhibiting a faster life history than native LP populations. As expected given the high fecundity and fast life history of HP guppies, differences in recruitment rates between immigrants/hybrids and native guppies seemed to drive the overall differences in fitness given that survival was fairly even across groups of different genetic ancestry (Fig. 5). However, interestingly, recruitment of F_2_ and backcrossed hybrids exceeded that of immigrants (Fig. 6). In this case it is possible that selection favored individuals with a combination of high fecundity traits from the source population and other locally adapted traits from the recipient LP population.

### Conservation relevance of genetic rescue in guppies

Our detailed characterization of genetic rescue in Trinidadian guppies helps fill important gaps for understanding how gene flow could be used to manage imperiled populations and species. Frankham et al. (2011) provides a flow chart of recommendations for avoiding outbreeding depression, but some of its questions such as ‘do substantial environmental differences exist?’ present major remaining uncertainties. In our system, predation level and density dependent competition are primary drivers of local adaptation in guppies (Reznick et al. 2001; Travis et al. 2014). The populations brought into contact by the introduction experiments were phenotypically adapted to opposite ends of these ecological gradients (Torres‐Dowdall et al. 2012). Yet our results suggest that adaptive divergence does not necessarily prevent fitness benefits from gene flow.

Our study also illustrated how different rates of migration and gene flow can lead to drastic differences in genetic composition of the population. Unlike most management scenarios, we could not control the rate of immigration and gene flow into recipient populations. Differences in the location of introduced and recipient populations led to substantial differences between streams in the rate of gene flow. Over the first 17 months, we estimate that Taylor received an average of 182 migrants per generation, while Caigual received an estimated average of four migrants per generation. Overall, both streams experienced substantial and sustained increases in population size, regardless of these differences in migration rate. However, from a conservation standpoint, the lower migration rate in Caigual led to a more ideal outcome where the increase in population size was mostly due to success of the hybrids and pure native genotypes were maintained in the population. In contrast, high migration into Taylor led to a dramatic decline of the pure native genotype, which may have caused the loss of potentially important local alleles. Determining the appropriate level of gene flow to prevent inbreeding without swamping local adaptation is a high priority goal for conservation biologists. The classic rule of thumb is one‐migrant‐per‐generation (Spieth 1974; Mills and Allendorf 1996), yet complexities inherent to natural populations can undermine the usefulness of this rule (Vucetich and Waite 2000; Wang 2004). For example, assumptions of equal selective advantage among genotypes, similar demographic attributes among immigrants and residents, and census sizes equal to effective population sizes are typically violated in imperiled natural populations (Mills and Allendorf 1996). In our case, an understanding of the environment (i.e., immigrants are likely to survive, given the low predation) and fast life history of immigrants (i.e., immigrants are likely to have higher fecundity than natives) might have led us to the *a priori* conclusion that few migrants per generation (<10) would be sufficient to induce genetic rescue, as confirmed by the results from Caigual.

## Concluding remarks

Understanding the genetic factors that underlie demographic responses will improve our ability to manage connectivity and maintain healthy populations in the wild. The scenario we studied, where immigrants are adaptively divergent and the resident population has low genetic diversity, mimics a common situation faced by managers deciding whether to augment endangered populations. Although many questions remain, our results suggest that adaptive divergence should not, in itself, preclude the use of assisted gene flow for inducing fitness benefits, and also that low levels of migration can result in genetic rescue without replacing the native genetic signature. Future studies that employ genome‐wide markers (e.g., SNPs) will aid in understanding the genomic basis of genetic rescue and hopefully provide additional insight on the optimal number and type of migrants, and how neutral versus selected loci respond to selection and gene flow. Ultimately, sufficient habitat is necessary for long‐term persistence, but genetic rescue may provide a demographic buffer that allows populations to persist through environmental disturbances, as well as the genetic variation needed to adapt to a changing world.

## Data archiving statement

Data available from the Dryad Digital Repository: http://dx.doi.org/10.5061/dryad.rn262

## Supporting information


**Appendix S1.** Development and characterization of 12 microsatellite loci for the Trinidadian guppy (*Poecilia reticulata*).
**Appendix S2.** Modeling detection probability with capture‐mark‐recapture data.
**Table S1.** Characteristics of 12 microsatellite loci in *Poecilia reticulata*.
**Table S2.** Model selection results for detection probability (*P*) using the Pradel model.
**Table S3.** Model selection results for population growth rate (*λ*) using Pradel models with the full capture‐mark‐recapture dataset with 29 capture occasions.
**Table S4.** Model selection results for survival (*ϕ*) and recruitment (*f*) using Pradel models with the genotyped subset of capture‐mark‐recapture data with 17 capture occasions.
**Table S5.** Parameter estimates and 95% confidence intervals from the highest supported models for survival (*ϕ*) and recruitment (*f*).
**Figure S1.** Distribution of average overall performance scores (Vähä and Primmer 2006) as a function of the threshold value used to assign individuals to genotypic classes in NEWHYBRIDS.
**Figure S2.** Monthly estimates of detection probability (*P*) from the most parsimonious Pradel model (*P*(stream × month), Table S2).
