# Supplementary material for: Gene flow from an adaptively divergent source causes rescue through genetic and demographic factors in two wild populations of Trinidadian guppies
Source: Evol Appl. 2016 Feb 4;9(7):879–91. doi: 10.1111/eva.12356 (PMC4947150; doi:10.1111/eva.12356)

**Appendix I**

**Development and characterization of 12 microsatellite loci for the Trinidadian guppy (*Poecilia reticulata*)**

We used independent, neutral, and variable microsatellite loci to determine changes in genetic diversity and identify unmarked fish as recruits from the native Caigual and Taylor populations, new HP immigrants, or hybrids. We first screened 80 of 126 microsatellite loci that had been developed for this species prior to our work (Paterson et al., 2005; Shen et al., 2007; Watanabe et al., 2003, 2004). We did not find adequate polymorphism using these loci. For example, 42 out of 58 loci that amplified in both native populations were homozygous and fixed for the same allele. Due to the lack of genetic variation found in pre-existing loci, we developed a new microsatellite library for this study using Illumina sequencing in collaboration with the Evolutionary Genomics Core Facility at Cornell University.

Genomic DNA was purified from muscle tissue of five native Caigual and five native Taylor guppies that were sampled prior to gene flow using Qiagen DNeasy Blood and Tissue Kits. DNA was eluted with 100 μl AE buffer and concentration was determined on a Qubit 2.0 fluorometer. Each DNA sample was given one of two barcodes based on population (Caigual or Taylor) in order to filter loci for those with allelic variants in both populations. The following steps were thus completed using two sets of pooled DNA from five individuals per population. Genomic DNA (50-100 ng) was digested with the restriction enzymes *AluI*, *RsaI*, and *Hpy166II*, in three separate reactions. After heat inactivation of the restriction enzymes equal amounts of the three digests were combined in a single tube and the blunt ends were adenylated (+A) with Klenow (exo-) and dATP. After heat inactivation of the Klenow (exo-), the reactions were supplemented with ATP to 1 mM and an Illumina Y-adaptor was ligated with T4 DNA ligase. Fragments were enriched for microsatellites by hybridization to 3'-biotinylated repeat probes (representing two unique dimers, five unique trimers, four unique tetramers and two unique pentamers). Enriched genomic fragments were captured by streptavidin-coated magnetic beads, and fragments were amplified with Platinum Taq polymerase and a pair of Illumina primers (one universal, one index). PCR products were analyzed on an agarose gel and quantified with a Qubit 2.0 fluorometer. Equal amounts of each library were pooled and fragments 300-600 basepairs (bp) were recovered with Ampure beads. Libraries were submitted to the Sequencing and Genotyping Facility at the Cornell Life sciences Core

Laboratory Center (CLC) for 2 x 250 paired end sequencing on an Illumina MiSeq.

Barcode-sorted reads were trimmed of adapter sequences and assembled with SeqMan NGen v4.1.0.147. Consensus files and singleton reads were exported as fasta files and simple repeats and associated genotyping primers were summarized with msatcommander v1.0.3. For primer design we chose a product size range of 150-450 bp, primer minimum, optimum, and maximum sizes of 22,23, and 24 bases respectively. Minimum, optimum, and maximum annealing temperatures were set respectively to 58, 60, and 62 °C.

A total of 116 loci were discovered after filtering the total set to include only tetramers found in both Caigual and Taylor populations and had variable repeat lengths in at least one population. We conducted an initial screening for variability on 36 loci using a "universal tag" approach (Schuelke, 2000). PCR amplifications were carried out in 12.5 μl reactions containing 8.4 μl H_2_O, 1.6 μl 10x ABI buffer I with added MgCl_2_, 0.25 μl dNTPs, 0.1 μl BSA, 0.28 μl reverse primer (10 μM), 0.15 μl forward primer (10 μM), 0.15 μl dye-labeled M13 primer (10 μM), 0.06 μl AmpliTaq DNA polymerase, and 1.5 μl DNA. All reactions were performed using thermocycling conditions of: 95 °C for 3 min; 40 cycles at 95 °C for 30 s, 60 °C for 30 s, 72 °C for 30 s; 8 cycles at 94 °C for 30 s, 53 °C for 30 s, 72 °C for 45 s; and a final extension at 72 °C for 10 min. PCR products were mixed with HiDi formamide and LIZ ladder (500 GeneScan) and read on an ABI 3730 genetic analyzer (Life Sciences Core Laboratories at Cornell University). Fragment sizes were manually confirmed using GENEMARKER^®^ v1.91 (SoftGenetics, LLC, State College, PA, USA).

Sixteen out of 36 loci amplified and were polymorphic in seven individuals (three Caigual, four Taylor). We next tested these 16 loci at 20 additional individuals from each of Caigual and Taylor native populations using the same PCR protocol described above. Conformity of genotype proportions to Hardy-Weinberg equilibrium (HWE) and linkage disequilibrium (LD) was tested using GENEPOP v4.2 (Raymond and Rousset, 1995). Microsatellite loci were examined for evidence of null alleles and scoring error due to stutter or large allele dropout using MICROCHECKER v2.2.3 (van Oosterhout et al., 2006).

We recovered a final set of 12 loci that fit HWE expectations, did not show evidence for LD or null alleles, and were variable in native Caigual and Taylor populations. We divided these 12 loci into three panels of four loci each for multiplexing PCR reactions. Dye-labeled forward primers were ordered using 6-FAM from Integrated DNA Technologies and the Applied Biosystems G5 dye set (PET, VIC, NED). We performed multiplexed PCR reactions on the remainder of individuals using the QIAGEN Type-it Microsatellite PCR kits. These reactions contained 4 μl of H_2_O, 6.25 μl of Type-it Master Mix, 0.1 μl of BSA, 1.25 μl of the primer mix (each primer at 2 μM), and 1 μl of DNA. All reactions were performed using thermocycling conditions of: 95 °C for 10 min; 35 cycles at 95 °C for 30 s, 60 °C for 30 s, 72 °C for 30 s; and a final extension at 60 °C for 30 min. Fragment analysis was performed using the same protocol described above. We confirmed that peaks obtained from multiplex reactions corresponded to those from single-locus PCRs.

**Simulations to optimize genetic class assignments in NEWHYBRIDS**

We assessed the power of NEWHYBRIDS to correctly assign individuals to genotypic classes by analyzing a set of simulated (i.e., genotypes of known genetic ancestry). To generate simulated data, we used twenty known pure native Caigual individuals sampled prior to gene flow, and twenty individuals known to originate from the introduction site. From these pure "parental" genotypes, we generated 100 genotypes in HYBRIDLAB 1.0 (Nielsen et al., 2006) for each of the following genotypic classes: Native, Immigrant, F1, F2, F1xNative, F1xImmigrant, for a total of 600 individuals. We then used this simulated dataset in NEWHYBRIDS with default settings for 100,000 MCMC iterations and discarding the first 10,000 as burn-in. We repeated this process using twenty pure Taylor individuals as one of the parental populations.

NEWHYBRIDS returns posterior probability values that represent each individual's probability of belonging to one of the six genotypic classes. To optimize the posterior probability threshold value for my dataset, we calculated efficiency and accuracy scores and obtained an "overall performance score" across all simulated genotypes, using a range of threshold values (0.5-0.95), as recommended by (Vähä and Primmer, 2006). An optimized performance score should maximize the number of identified members of a genotypic class while maintaining high accuracy. Using NEWHYBRIDS results from simulated data, the posterior probability threshold that optimized overall performance score (averaged across each genotypic class) and had the lowest standard deviation was 0.50 (Figure S1). We used this threshold for classifying individuals into genetic groups as described in the main text.

**Appendix II**

**Modeling detection probability with capture-mark-recapture data**

We estimated detection probability (*p*) by fitting a Pradel model in Program MARK v8.0 to the full 29 months of individual capture histories. We expected detection probability to vary by stream due to differences in pool structure and flow and by month due to seasonal differences in flow. We did not have a priori reasons to expect differences in detection probability between sexes or among genetic classification groups, and thus did not include them as factors. All models included the most general structure for survival (*ϕ*) and population growth rate (*λ*); a three-way interaction among sex, stream, and month. We compared the most complex structure for *p,* which included an interaction between stream and month to simplified structures that included single factors of stream and month and the constant model (Table S2). Models were fit with Maximum Likelihood and compared using Akaike's Information Criterion adjusted for sampled size (AICc) and AICc weights.

The top-ranking model, with 100% of the weight of evidence, supported the most general detection structure with an interaction between stream and month (Table S2). Overall, detection probability was high. Temporal variation in capture probability was consistent with seasonal changes in water level and flow (Figure S2).

LITERATURE CITED

Nielsen, E.E., Bach, L.A., and Kotlicki, P. (2006). hybridlab (version 1.0): a program for generating simulated hybrids from population samples. Mol. Ecol. Notes *6*, 971–973.

Van Oosterhout, C., Weetman, D., and Hutchinson, W.F. (2006). Estimation and adjustment of microsatellite null alleles in nonequilibrium populations. Mol. Ecol. Notes *6*, 255–256.

Paterson, I.G., Crispo, E., Kinnison, M.T., Hendry, A.P., and Bentzen, P. (2005). Characterization of tetranucleotide microsatellite markers in guppy ( Poecilia reticulata ). Mol. Ecol. Notes *5*, 269–271.

Raymond, M., and Rousset, F. (1995). GENEPOP (version 1.2): population genetics software for exact tests and ecumenicism. J. Hered. *86*, 248–249.

Schuelke, M. (2000). An economic method for the fluorescent labeling of PCR fragments. Nat. Biotechnol. *18*, 233–234.

Shen, X., Yang, G., and Liao, M. (2007). Development of 51 genomic microsatellite DNA markers of guppy (Poecilia reticulata) and their application in closely related species. Mol. Ecol. Notes *7*, 302–306.

Vähä, J.-P., and Primmer, C.R. (2006). Efficiency of model-based Bayesian methods for detecting hybrid individuals under different hybridization scenarios and with different numbers of loci. Mol. Ecol. *15*, 63–72.

Watanabe, T., Yoshida, M., Nakajima, M., and Taniguchi, N. (2003). Isolation and characterization of 43 microsatellite DNA markers for guppy (Poecilia reticulata). Mol. Ecol. Notes *3*, 487–490.

Watanabe, T., Nakajima, M., Yoshida, M., and Taniguchi, N. (2004). Construction of six linkage groups in the guppy (Poecilia reticulata). Anim. Genet. *35*, 147–148.

**Table S1.** Characteristics of 12 microsatellite loci in *Poecilia reticulata*.

|  |  |  |  |  |  | **All** | **native**  **Caigual** | | | **native**  **Taylor** | | |
| --- | --- | --- | --- | --- | --- | --- | --- | --- | --- | --- | --- | --- |
| **Locus** | **Panel** | **Dye** | **Repeat motif** | **Forward primer** | **Size range (bp)** | **N_A_** | **N_A_** | ***H*_O_** | ***H*_E_** | **N_A_** | ***H*_O_** | ***H*_E_** |
| Prgf006 | 2 | FAM | AGAT | **F:**AAGAAACAAAGCCAGTCCAACAC  **R:** TGCCTCTGGTTGGATTTATTGAC | 161-269 | 20 | 5 | 0.48 | 0.45 | 4 | 0.41 | 0.52 |
| Prgf008 | 1 | PET | AGAT | **F:**CATGAGGGTCTGTTCTTTCCATG  **R:** TCTCTTACGCCAGATAGATCGATC | 193-353 | 17 | 5 | 0.43 | 0.37 | 4 | 0.41 | 0.46 |
| Prgf021 | 1 | VIC | AGAT | **F:**CAGGTTGCTGTCTTGTTGCTTC  **R:** TGTCGATGTTGTCTACTGCAAAG | 208-284 | 18 | 7 | 0.66 | 0.79 | 6 | 0.63 | 0.76 |
| Prgf025 | 3 | VIC | AAAG | **F:**TCGCTAAGCAACGTATGAAACAC  **R:** ACTAATACGAGGGAAGTGGAAGG | 228-344 | 20 | 7 | 0.60 | 0.73 | 5 | 0.82 | 0.74 |
| Prgf027 | 1 | NED | AGAT | **F:**GTGGATGCAGTGTCTCTATCATG  **R:** TTGTCACTGTTTAAGCATCTGGG | 188-260 | 18 | 11 | 0.71 | 0.83 | 3 | 0.30 | 0.31 |
| Prgf034 | 1 | FAM | AAAG | **F:**CCCATTCACCCTATTTCCCAAAG  **R:** GCCCACTCCCTTTCCGTAATATC | 253-341 | 20 | 4 | 0.07 | 0.12 | 3 | 0.19 | 0.24 |
| Prgf038 | 2 | PET | AGAT | **F:**GGTCACGTGGTTTGGAAATGTC  **R:** AAAGCATCCCGACAGTATGATTC | 174-298 | 17 | 5 | 0.59 | 0.63 | 4 | 0.26 | 0.24 |
| Prgf039 | 3 | NED | AAAC | **F:**TCCCTTTCCTTGCTGAAGTTTAAG  **R:** ACAAAGGTCTGCATAATTGTGATG | 208-282 | 10 | 2 | 0.19 | 0.23 | 2 | 0.11 | 0.11 |
| Prgf040 | 2 | NED | AGAT | **F:**AGCATTGTTAGCATCACAGACAG  **R:** ACAGCCACCAATTAAGAAACCAG | 175-235 | 15 | 2 | 0.26 | 0.32 | 4 | 0.19 | 0.21 |
| Prgf042 | 2 | VIC | AGAT | **F:**ACATAACATTCCTTTAGTGCACG  **R:** AGGAGCAATAAGAAGAAGGGTTC | 170-230 | 10 | 3 | 0.20 | 0.19 | 2 | 0.37 | 0.35 |
| Prgf043 | 3 | PET | ATCC | **F:**CCTTTCCCTGTGGTGAATATTGG  **R:** AGTCTTTGCCTCCCTACTTAGAC | 194-280 | 17 | 3 | 0.31 | 0.27 | 2 | 0.22 | 0.31 |
| Prgf053 | 3 | FAM | ATCC | **F:**CTGTACTTTGAAGCCACCCATC  **R:** GTTCATCTGCGTTCCAAGGATC | 114-244 | 12 | 3 | 0.36 | 0.37 | 5 | 0.56 | 0.56 |

**Table S2.** Model selection results for detection probability (*p*) using the Pradel model. Model structures were ranked using quasi-Akaike Information Criteria corrected for sample size (AIC_c_). Relative AIC_c_ (ΔAIC_c_), Akaike weight (*w*), number of parameters (*K*), and deviance are reported. All reported model structures were run with the most general model structure in the survival and population growth rate parameters: *ϕ*(stream × sex × month) *λ*(stream × sex × month). The model with constant detection probability (.) was compared to models with variation in stream, month, and additive and interactive interactions between stream and month.

| ***p* model structure** | **AIC_c_** | **ΔAIC_c_** | **w** | **K** | **Deviance** |
| --- | --- | --- | --- | --- | --- |
| Stream × month | 96492 | 0 | 1 | 238 | 6634 |
| Month | 96583 | 91 | 0 | 215 | 6772 |
| Stream | 96699 | 207 | 0 | 194 | 6931 |
| . | 96706 | 214 | 0 | 193 | 6939 |

**Table S3.** Model selection results for population growth rate (*λ*) using Pradel models with the full capture-mark-recapture dataset with 29 capture occasions. Model structures were ranked using Akaike Information Criteria corrected for sample size (AIC_c_). Relative AIC_c_ (ΔAIC_c_), Akaike weight (*w*), number of parameters (*K*), and deviance are reported. All reported model structures were run with the best supported model structure for survival: *ϕ*(stream × sex × month), and detection probability: *p*(stream × month).

| ***λ* model structure** | **AIC_c_** | **ΔAIC_c_** | **w** | **K** | **Deviance** |
| --- | --- | --- | --- | --- | --- |
| Stream × sex × month | 96492 | 0 | 1 | 238 | 6634 |
| Stream × month | 96606 | 113 | 0 | 190 | 6845 |
| Sex × month | 96752 | 260 | 0 | 193 | 6985 |
| Month | 96865 | 372 | 0 | 169 | 7147 |
| Stream × sex | 97705 | 1213 | 0 | 150 | 8025 |
| Stream | 97712 | 1220 | 0 | 148 | 8036 |
| Sex | 97755 | 1263 | 0 | 148 | 8079 |
| . | 97759 | 1267 | 0 | 147 | 8085 |

**Table S4.** Model selection results for survival (*ϕ*) and recruitment (*f*) using Pradel models with the genotyped subset of capture-mark-recapture data with 17 capture occasions. Model structures were ranked using Akaike Information Criteria (AIC_c_). Relative AIC_c_ (ΔAIC_c_), Akaike weight (*w*), number of parameters (*K*), and deviance are reported. All reported model structures were run with temporal variation in detection probability: *p*(month). Survival models were run with the three-way interaction on recruitment: *f*(Gen × sex × stream). Recruitment models run with the three-way interaction on survival: *ϕ*(Gen × sex × stream).

| ***ϕ* model structure** | **AIC_c_** | **ΔAIC_c_** | **w** | **K** | **Deviance** |
| --- | --- | --- | --- | --- | --- |
| Gen × sex × stream | 24580 | 0 | 0.67 | 64 | 4225 |
| Sex × stream | 24581 | 1 | 0.33 | 44 | 4267 |
| Gen × stream | 24606 | 26 | 0 | 52 | 4276 |
| Stream | 24618 | 38 | 0 | 42 | 4308 |
| Gen × sex | 24954 | 374 | 0 | 52 | 4624 |
| Gen | 24990 | 410 | 0 | 46 | 4672 |
| Sex | 25007 | 427 | 0 | 42 | 4697 |
| . | 25055 | 475 | 0 | 41 | 4747 |
| ***f* model structure** | **AIC_c_** | **ΔAIC_c_** | **w** | **K** | **Deviance** |
| Gen × stream | 24571 | 0 | 0.99 | 52 | 4241 |
| Gen × sex × stream | 24580 | 9 | 0.01 | 64 | 4225 |
| *Gen* | 24634 | 63 | 0 | 46 | 4316 |
| Gen × sex | 24635 | 64 | 0 | 52 | 4305 |
| Sex × stream | 25534 | 964 | 0 | 44 | 5220 |
| Stream | 25573 | 1002 | 0 | 42 | 5263 |
| Sex | 25950 | 1380 | 0 | 42 | 5640 |
| . | 26001 | 1430 | 0 | 41 | 5693 |

**Table S5.** Parameter estimates and 95% confidence intervals from the highest supported models for survival (*ϕ*) and recruitment (*f*). The best supported model included the survival structure *ϕ*(Gen × sex × stream) and the recruitment structure *f*(Gen × stream) as reported in Table S4.

| **Stream** | **Sex** | **Group** | *ϕ* | **95% CI** | ***f*** | **95% CI** |
| --- | --- | --- | --- | --- | --- | --- |
| Caigual | F | native | 0.81 | (0.79,0.82) | 0.11 | (0.10,0.13) |
| Caigual | F | immigrant | 0.92 | (0.61,0.99) | 0.56 | (0.40,0.70) |
| Caigual | F | F_1_ hybrid | 0.86 | (0.82,0.90) | 0.52 | (0.46,0.59) |
| Caigual | F | F_2_ hybrid | 0.84 | (0.43,0.97) | 1.39 | (0.80,1.98) |
| Caigual | F | F_1_ x native backcross | 0.84 | (0.71,0.92) | 0.88 | (0.50,0.98) |
| Caigual | F | F_1_ x immigrant backcross | 0.90 | (0.79,0.95) | 0.92 | (0.47,0.99) |
| Caigual | M | native | 0.71 | (0.68,0.74) | 0.11 | (0.10,0.13) |
| Caigual | M | immigrant | 0.78 | (0.66,0.86) | 0.56 | (0.40,0.70) |
| Caigual | M | F_1_ hybrid | 0.80 | (0.74,0.85) | 0.52 | (0.46,0.59) |
| Caigual | M | F_2_ hybrid | 0.79 | (0.52,0.93) | 1.39 | (0.80,1.98) |
| Caigual | M | F_1_ x native backcross | 0.79 | (0.62,0.89) | 0.88 | (0.50,0.98) |
| Caigual | M | F_1_ x immigrant backcross | 0.82 | (0.70,0.90) | 0.92 | (0.47,0.99) |
| Taylor | F | native | 0.55 | (0.51,0.59) | 0.23 | (0.20,0.26) |
| Taylor | F | immigrant | 0.61 | (0.57,0.65) | 0.61 | (0.56,0.65) |
| Taylor | F | F_1_ hybrid | 0.57 | (0.50,0.63) | 0.47 | (0.42,0.53) |
| Taylor | F | F_2_ hybrid | 0.53 | (0.40,0.65) | 0.72 | (0.58,0.83) |
| Taylor | F | F_1_ x native backcross | 0.37 | (0.24,0.52) | 0.72 | (0.57,0.83) |
| Taylor | F | F_1_ x immigrant backcross | 0.55 | (0.46,0.65) | 0.82 | (0.71,0.90) |
| Taylor | M | native | 0.51 | (0.45,0.56) | 0.23 | (0.20,0.26) |
| Taylor | M | immigrant | 0.58 | (0.54,0.63) | 0.61 | (0.56,0.65) |
| Taylor | M | F_1_ hybrid | 0.51 | (0.43,0.59) | 0.47 | (0.42,0.53) |
| Taylor | M | F_2_ hybrid | 0.39 | (0.25,0.55) | 0.72 | (0.58,0.83) |
| Taylor | M | F_1_ x native backcross | 0.46 | (0.29,0.63) | 0.72 | (0.57,0.83) |
| Taylor | M | F_1_ x immigrant backcross | 0.57 | (0.49,0.66) | 0.82 | (0.71,0.90) |

**Figure S1.** Distribution of average overall performance scores (Vähä and Primmer, 2006) as a function of the threshold value used to assign individuals to genotypic classes in NEWHYBRIDS. We determined that a threshold of 0.5 was most appropriate based on the overall performance score and distribution of the data around the mean.
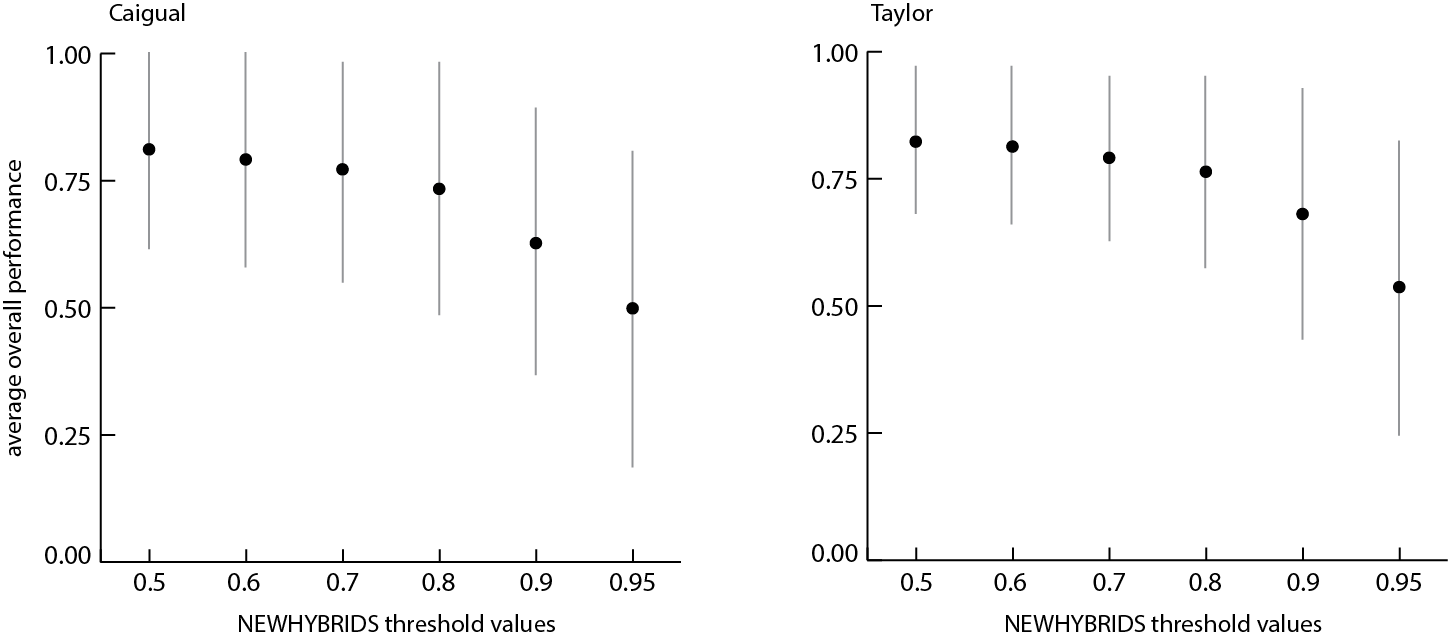


**Figure S2.** Monthly estimates of detection probability (*p*) from the most parsimonious Pradel model (*p*(stream×month), Table S2).


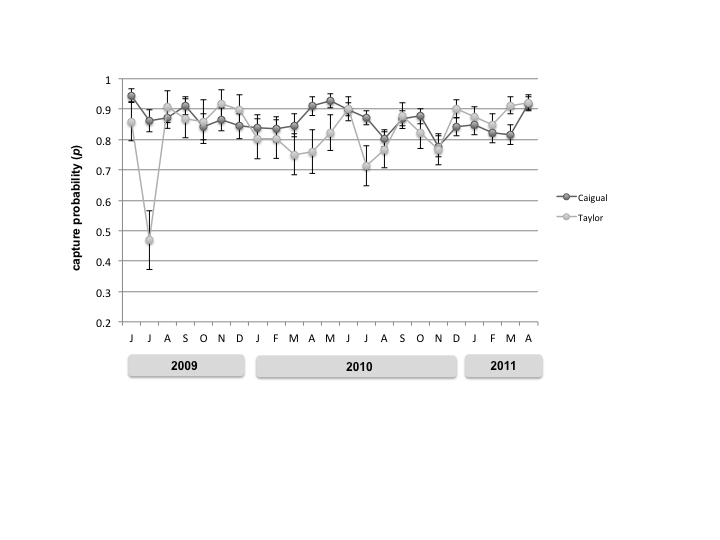

Supplement: Supplementary file 1 — Appendix S1. Development and characterization of 12 microsatellite loci for the Trinidadian guppy (Poecilia reticulata). Appendix S2. Modeling detection probability with capture‐mark‐recapture data. Table S1. Characteristics of 12 microsatellite loci in Poecilia reticulata. Table S2. Model selection results for detection probability (P) using the Pradel model. Table S3. Model selection results for population growth rate (λ) using Pradel models with the full capture‐mark‐recapture dataset with 29 capture occasions. Table S4. Model selection results for survival (ϕ) and recruitment (f) using Pradel models with the genotyped subset of capture‐mark‐recapture data with 17 capture occasions. Table S5. Parameter estimates and 95% confidence intervals from the highest supported models for survival (ϕ) and recruitment (f). Figure S1. Distribution of average overall performance scores (Vähä and Primmer 2006) as a function of the threshold value used to assign individuals to genotypic classes in NEWHYBRIDS. Figure S2. Monthly estimates of detection probability (P) from the most parsimonious Pradel model (P(stream × month), Table S2). [file EVA-9-879-s001.docx]
